# The STRING database in 2025: protein networks with directionality of regulation

**DOI:** 10.1093/nar/gkae1113

**Published:** 2024-11-18

**Authors:** Damian Szklarczyk, Katerina Nastou, Mikaela Koutrouli, Rebecca Kirsch, Farrokh Mehryary, Radja Hachilif, Dewei Hu, Matteo E Peluso, Qingyao Huang, Tao Fang, Nadezhda T Doncheva, Sampo Pyysalo, Peer Bork, Lars J Jensen, Christian von Mering

**Affiliations:** Department of Molecular Life Sciences, University of Zurich, Winterthurerstrasse 190, 8057 Zurich, Switzerland; SIB Swiss Institute of Bioinformatics, Amphipôle, Quartier UNIL-Sorge, 1015 Lausanne, Switzerland; Novo Nordisk Foundation Center for Protein Research, University of Copenhagen, Blegdamsvej 3B, 2200 Copenhagen N, Denmark; Novo Nordisk Foundation Center for Protein Research, University of Copenhagen, Blegdamsvej 3B, 2200 Copenhagen N, Denmark; Novo Nordisk Foundation Center for Protein Research, University of Copenhagen, Blegdamsvej 3B, 2200 Copenhagen N, Denmark; TurkuNLP Lab, Department of Computing, University of Turku, Vesilinnantie 5, 20014 Turku, Finland; Department of Molecular Life Sciences, University of Zurich, Winterthurerstrasse 190, 8057 Zurich, Switzerland; SIB Swiss Institute of Bioinformatics, Amphipôle, Quartier UNIL-Sorge, 1015 Lausanne, Switzerland; Novo Nordisk Foundation Center for Protein Research, University of Copenhagen, Blegdamsvej 3B, 2200 Copenhagen N, Denmark; Department of Molecular Life Sciences, University of Zurich, Winterthurerstrasse 190, 8057 Zurich, Switzerland; SIB Swiss Institute of Bioinformatics, Amphipôle, Quartier UNIL-Sorge, 1015 Lausanne, Switzerland; Department of Molecular Life Sciences, University of Zurich, Winterthurerstrasse 190, 8057 Zurich, Switzerland; SIB Swiss Institute of Bioinformatics, Amphipôle, Quartier UNIL-Sorge, 1015 Lausanne, Switzerland; Department of Molecular Life Sciences, University of Zurich, Winterthurerstrasse 190, 8057 Zurich, Switzerland; SIB Swiss Institute of Bioinformatics, Amphipôle, Quartier UNIL-Sorge, 1015 Lausanne, Switzerland; Novo Nordisk Foundation Center for Protein Research, University of Copenhagen, Blegdamsvej 3B, 2200 Copenhagen N, Denmark; TurkuNLP Lab, Department of Computing, University of Turku, Vesilinnantie 5, 20014 Turku, Finland; Structural and Computational Biology Unit, European Molecular Biology Laboratory, Meyerhofstrasse 1, 69117 Heidelberg, Germany; Max Delbrück Centre for Molecular Medicine, Robert-Rössle-Strasse 10, 13125 Berlin, Germany; Department of Bioinformatics, Biozentrum, University of Würzburg, Am Hubland, 97074 Würzburg, Germany; Novo Nordisk Foundation Center for Protein Research, University of Copenhagen, Blegdamsvej 3B, 2200 Copenhagen N, Denmark; Department of Molecular Life Sciences, University of Zurich, Winterthurerstrasse 190, 8057 Zurich, Switzerland; SIB Swiss Institute of Bioinformatics, Amphipôle, Quartier UNIL-Sorge, 1015 Lausanne, Switzerland

## Abstract

Proteins cooperate, regulate and bind each other to achieve their functions. Understanding the complex network of their interactions is essential for a systems-level description of cellular processes. The STRING database compiles, scores and integrates protein–protein association information drawn from experimental assays, computational predictions and prior knowledge. Its goal is to create comprehensive and objective global networks that encompass both physical and functional interactions. Additionally, STRING provides supplementary tools such as network clustering and pathway enrichment analysis. The latest version, STRING 12.5, introduces a new ‘regulatory network’, for which it gathers evidence on the type and directionality of interactions using curated pathway databases and a fine-tuned language model parsing the literature. This update enables users to visualize and access three distinct network types—functional, physical and regulatory—separately, each applicable to distinct research needs. In addition, the pathway enrichment detection functionality has been updated, with better false discovery rate corrections, redundancy filtering and improved visual displays. The resource now also offers improved annotations of clustered networks and provides users with downloadable network embeddings, which facilitate the use of STRING networks in machine learning and allow cross-species transfer of protein information. The STRING database is available online at https://string-db.org/.

## Graphical Abstract

**Graphical Abstract:**
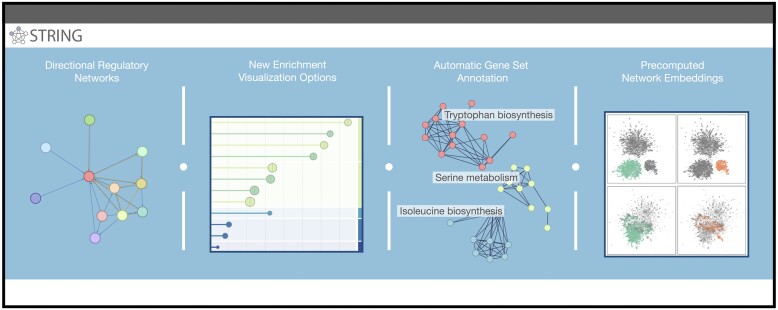

## Introduction

The function of every living cell is primarily governed by a complex network of interacting proteins, with each protein’s role determined not only by its molecular activities but also by its position within this network (1,2). Connected proteins work together to contribute to common biological processes through various interaction types, such as physical binding, genetic interactions and regulatory influences. These interactions can collectively be categorized as *functional associations*, which serve as fundamental operational units within biological systems. Unraveling protein networks in their various modalities remains a significant research focus. Consequently, numerous databases have been developed over the years to meet specific research needs. These range from carefully curated pathway databases such as Reactome (3) and KEGG (4), to databases of experimental interaction evidence curated from literature such as BioGRID (5), IntAct (6) and MINT (7), and to those centered on specific modes of interactions, including Complex Portal for protein co-complexes (8) and SIGNOR for regulatory interactions (9). Finally, composite databases such as STRING (10), GeneMANIA (11), FunCoup (12) and HumanNet (13) not only incorporate data from these sources but also employ an array of computational methods to predict additional associations, striving to provide the most comprehensive views of the interactome.

Among these databases, STRING is notable for its many sources of evidence, its robust scoring system, user-friendly interface and comprehensive suite of enrichment features. It is dedicated to assembling a broad set of associations among proteins for the complete proteomes of thousands of organisms across all domains of life. STRING charts interactions ranging from highly confident, well-documented associations to more speculative ones, which are crucial for exploratory and computational analyses. The scoring system ensures that data from diverse sources—including automatic text mining, high- and low-throughput experimental data, and computational predictions—are directly comparable and weighted consistently, regardless of their origin. Additionally, STRING extends and maps networks across species by predicting interologs, thereby broadening the scope of functional associations to encompass a diverse range of organisms. This includes uncultured novel bacterial species derived from metagenomic samples, and even unpublished proteomes uploaded by users.

The development of interaction databases has so far been a trade-off between detailing the exact mode of an interaction—whether physical or functional, signaling or structural, stable or transient—and maintaining a comprehensive set of interactions. Given the limitations of the available data and methodologies, STRING has historically focused on broadly defined functional associations, which provided the most useful set of interactions for proteome-wide analysis. While these networks continue to perform well in downstream tasks (14,15), they often lack fine-grained resolution, leaving more specialized databases better equipped to offer detailed insights into specific interactions if needed. However, the growing volume of data and advancements in text-mining technologies have since enabled the composite, general-purpose databases to catch up, offering more detailed maps of the interaction space. Recently, the STRING database introduced a co-complex (physical) interaction network mode, which details multi-protein assemblies. With the latest update, the database now also includes the largest set of regulatory (directed) interactions, highlighting the flow of information within cells. This major enhancement greatly expands the analytical capabilities of the database, enabling new types of studies that were not previously possible with nondirected interaction networks.

Interaction and pathway databases are frequently used to gain insights into the functional context of individual proteins or to understand the biological organization of an entire protein dataset. Many of these databases enhance the interpretation of protein datasets beyond simple interaction annotations by incorporating an enrichment analysis toolset. This type of analysis involves comparing observed data against expected distributions, enabling researchers to identify statistically significant features or patterns. Enrichment tools typically utilize established ontologies and annotations, such as Gene Ontology (16), MSigDB hallmark sets (17) or OMIM (18). A few databases also leverage their annotated datasets to help understand the functional context of a user’s input. For example, KEGG (4) employs pathway and module abstractions to organize genes into datasets for overrepresentation analysis, while Reactome maps interconnected proteins into hierarchically nested modules. Uniquely, STRING uses an unsupervised procedure to hierarchically precluster its entire proteome-wide networks into functional modules. Incorporating these network-derived gene sets into the enrichment analysis facilitates the identification of novel modules, especially in areas of the proteome where manual curation has been less comprehensive so far.

## Database content

The basic interaction scope in STRING is that of a ‘functional association’ between pairs of proteins. A functional association is defined as a contribution of two non-identical proteins to a common function (19,20). This can take many forms; functionally associated proteins can be in physical proximity to each other, regulate each other, exhibit genetic epistasis or even work antagonistically (as long as this occurs in the context of a common function). For the purpose of defining functional associations, the concept of a common function is crucial, but difficult to define—it should broadly be thought of as corresponding to the notion of a ‘pathway’ or ‘function module’. In practice, STRING roughly follows the functional granularity of the pathway maps in the KEGG database (4).

From the set of all functional associations in STRING, subsets are derived that are more specifically annotated with regard to their mechanism of association. Currently, two such more specific subsets are implemented: ‘physical’ and ‘regulatory’. The physical mode refers to pairs of proteins that either bind directly or are at least subunits of the same complex (21). The regulatory mode refers to associated protein pairs that are known to regulate each other’s activity in at least one direction. This mode is described in more detail further below; for the first time in STRING, such connections are annotated not only with confidence scores but also with a directionality.

All protein–protein associations in STRING are annotated with ‘confidence scores’. These scores are fully precomputed; they scale between 0 and 1 and describe the estimated likelihood of a postulated association being correct, given the available evidence. Separate confidence scores are provided for physical and regulatory modes—these scores provide estimates of the likelihood that the proposed association is taking place and is indeed of the postulated type. The two specific network modes are fully consistent with the functional network, such that if an interaction is present in either the physical or regulatory network, it will, by definition, also be present in the full functional association network (with an equal or higher confidence score). To compute the various confidence scores, the available evidence supporting a given interaction is first separated into ‘evidence channels’, by the type of evidence. For the basic functional association confidence score, seven such channels are used. These include three that are based on genomic context predictions—neighborhood, fusion and gene co-occurrence—as well as channels dedicated to co-expression, experimental data, curated databases and text mining.

The *genomic context channels* focus on associations inferred from genome sequences alone, detecting evolutionary patterns such as gene proximity, fusion events and co-occurrence across different organisms. For instance, the *neighborhood channel* assigns an association score to gene pairs that are located close to each other on the chromosome in the same orientation (in prokaryotic genomes) (22). The *fusion channel* identifies open reading frames that result from gene fusion events (23), while the *co-occurrence channel* examines whether genes have a nontrivial but shared distribution across genomes (24), implying a shared history of horizontal transfers, losses or duplication events and thus likely related functions. The *co-expression channel* compiles data from gene expression studies, analyzing both transcript and protein abundances across various conditions. By comparing expression profiles, it identifies gene pairs with similar expression patterns, suggesting functional linkage (10,25). The *experiments channel* aggregates interaction evidence from laboratory assays, including biochemical, biophysical and genetic assays. Data are imported from primary repositories such as BioGRID (5) and the IMEx consortium (26), and the confidence scores are estimated by globally benchmarking the accuracy of annotated experimental techniques, as well as within-dataset performance and consistency for the case of high-throughput experiments (10). The *database channel* is based on well-described, curated protein–protein associations from expert-compiled resources, such as KEGG (4), Reactome (3) and Gene Ontology Complexes (16). These resources provide well-established pathways, complexes and functional relationships, offering a high level of reliability. Unlike other channels, the database channel assigns a uniformly high confidence score to associations, reflecting their established nature. Finally, the *text-mining channel* utilizes a large corpus of scientific literature, including PubMed abstracts and full-text articles, to identify co-mentions of protein names (27). By statistically analyzing the frequency of these co-mentions in various textual contexts, this channel uncovers potential associations that may not be evident from structured data alone. In addition, for the more specific association modes, dedicated large language models are employed to detect sentences supporting either physical (21) or regulatory associations (see below).

For each evidence channel, the available interaction evidence is translated into a confidence score by first quantifying the evidence using channel-specific metrics and then converting these metrics into likelihoods using calibration curves derived from prior knowledge (from pathway-map memberships in KEGG). For the more specific association modes ‘physical’ and ‘regulatory’, only channels and evidence that are applicable to these modes are considered. After this, all channel scores that have been computed for a given protein pair in a given organism are transferred onto related protein pairs in other organisms, based on the ‘interolog’ concept (27,28). Lastly, a final, combined confidence score is computed by integrating the channel-specific subscores probabilistically, under the assumption that evidence in different channels is largely independent. Users of STRING can directly rely on this combined score for browsing and filtering networks, or they can alternatively customize their analyses by enabling or disabling specific channels separately, after which the combined score is recomputed according to their chosen settings.

All primary evidence underlying a given interaction can be inspected interactively, in dedicated evidence viewers online. Furthermore, accessory information is available for each protein, such as its three-dimensional structure, domain composition, annotation and cross-references. Apart from its core protein-network functionality, STRING also implements features that allow extensive analysis of uploaded user data. These include functional enrichment detection (29), experimental bias detection (21), homology searches, and clustering and organizing large query protein sets. All data contained in STRING are freely available for download, under a Creative Commons BY 4.0 license. Apart from its website, STRING can also be accessed via a dedicated Cytoscape plugin (30), through an application programming interface (API) (31), as well as through an R/Bioconductor package (https://www.bioconductor.org/packages/release/bioc/html/STRINGdb.html).

## Regulatory networks

Molecular networks, like those available through STRING, have become invaluable tools in biomedical research, offering powerful insights into how molecules work together within cells. The functional association networks, while useful for many applications, do not specify the interaction type (e.g. complex formation or transcriptional regulation) nor the direction of interaction (i.e. who regulates whom). Constructing networks with more detailed interaction types and directions can significantly expand their utility, particularly for building mathematical models of biological systems or for making logical inferences. The directionality of interactions is essential for interpretation of omics data, which typically reveals more about downstream effects of conditions such as diseases than it does about the upstream events that led to it and thus could be targeted therapeutically.

To incorporate interactions with specific directionality, sign and type into STRING, we leveraged the novel dataset and deep learning-based relation extraction method described in RegulaTome (32). The RegulaTome corpus provides a rich and diverse dataset that includes 16 961 relations between 54 951 entities annotated across over 2500 documents. This corpus is utilized to train a deep learning-based method used to annotate and extract the regulatory events throughout the entire available literature.

On top of undirected physical interactions—which are already included in STRING—the following types of directed interactions can be extracted using the system developed for RegulaTome: *Regulation*, with the signed subtypes of *Positive Regulation* and *Negative Regulation, Regulation of Gene Expression, Regulation of Degradation* and *Catalysis of Post-Translational Modifications*, covering six subtypes: *Catalysis of Small Protein Conjugation, Catalysis of Small Protein Removal, Catalysis of Phosphorylation, Catalysis of Dephosphorylation, Catalysis of Small Molecule Conjugation* and *Catalysis of Small Molecule Removal*. To enhance the interoperability of the annotations, these chosen relation types align with Gene Ontology (32).

To identify and classify a wide array of interaction types, we fine-tuned the RoBERTa-large-PM-M3-Voc language model, a transformer-based model well suited for biological tasks. This model was trained on the RegulaTome dataset for multi-label extraction of the directed, typed and signed interactions mentioned above, and achieved an average *F*_1_ score of 73.5% (with a precision of 75.2% and a recall of 71.8%) on a held-out test set, although the exact performance varied across different types of relationships. This level of accuracy is substantial, considering the complexity of the underlying task. Using this model, we processed over 1.2 billion sentence-level pairs extracted from all available PubMed abstracts and PMC Open Access full-text documents, and assigned 3.5% of them (∼43 million, of which ∼18 million in human) with at least one positive label indicating directed (72.9%) or signed (33.1%) relationships among the proteins. Furthermore, we made use of the existing curated knowledge of regulatory interactions in the *database* channel, by parsing regulatory information from the SIGNOR, KEGG and Reactome databases.

To integrate these interactions into STRING, we followed a similar score aggregation and benchmarking approach as for physical interactions (21). Here, we perform benchmarking in five categories for which we can derive gold standard datasets of known human regulatory interactions from SIGNOR: *regulation, upregulation, downregulation, transcriptional regulation* and *phosphorylation*. The resulting calibration functions are then applied to extracted regulations of all types to produce the final regulatory confidence scores.

**Figure 1.**
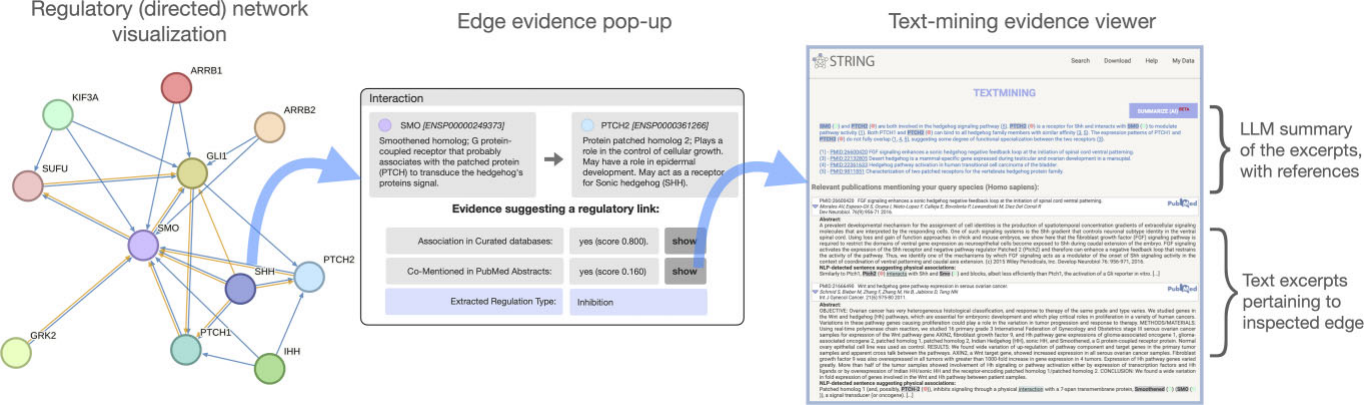
Illustration of the new ‘regulatory network’ mode in STRING, where the network edges visually indicate the direction, confidence and sources of each regulatory interaction. Clicking on an edge within the network brings up a pop-up window with a detailed overview of the available evidence and the annotated type of regulation. Users can further explore the data behind the interactions by accessing the specific evidence viewers linked within the pop-up, including the text-mining evidence viewer. This viewer presents excerpts from literature pertaining to the inspected interactions, as well as an automatically generated large language model summary of the presented excerpts.

The new regulatory network has been seamlessly integrated into the existing STRING framework, complementing the ‘functional’ and ‘physical’ network types. Directional edges within this network are visually represented by arrows, depicting both bidirectional and unidirectional relationships. The network visualization can show varying confidence levels between directions (in the ‘confidence’ view) or the specific directionality of the sources (in the ‘evidence’ view). The user interface retains its intuitive design, enabling users to access the type of regulatory event and the evidence for the interaction by clicking on the edge (Figure 1). Additionally, all API functions have been updated to fully support the new network type, which can be accessed by specifying the parameter *network_type*=*regulatory* in the API call.

**Figure 2.**
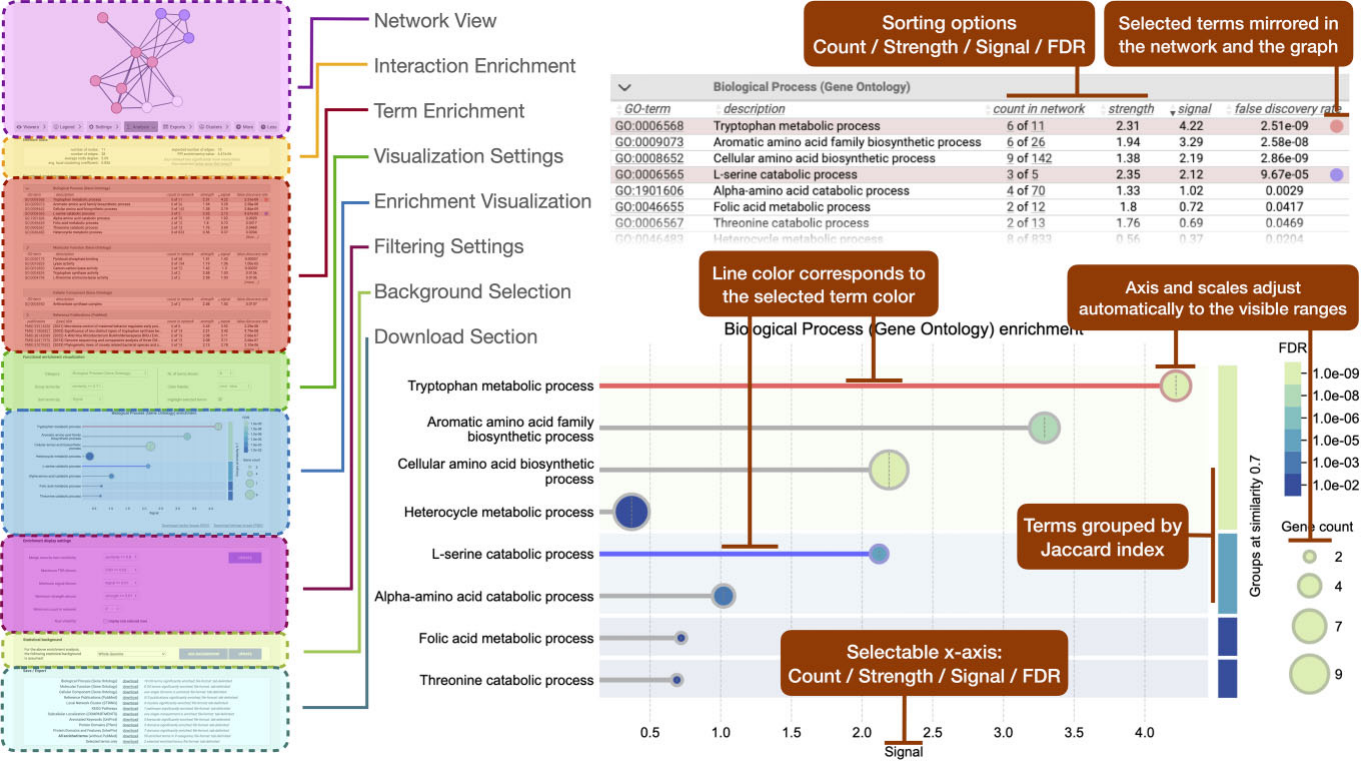
Enrichment analysis interface from the STRING database website. Left: Schematic of the enrichment analysis tab highlighting various sections of the webpage. Top right: A zoomed-in view of the analysis table with two user-highlighted terms (colored lines). Bottom right: A visualization of the enrichment depicting the two terms highlighted in corresponding colors.

## Expanded co-expression networks

The *co-expression* channel in STRING identifies genes with similar expression profiles across various tissues and conditions, revealing their involvement in shared biological processes. It compiles data from large-scale gene expression studies, analyzing transcript and protein levels to detect gene pairs with coordinated expression. This method highlights potential functional relationships between genes. Importantly, the analysis is free from study bias, as it directly uses raw omics data instead of relying on manually curated or experimental datasets, providing a more precise and objective view of gene interactions.

For the upcoming version 12.5, we are expanding the *co-expression* channel by generating additional networks through the application of FAVA (Functional Association of Variational Autoencoders) (25) on a wider range of single-cell RNA sequencing data. Specifically, we are incorporating single-cell data from the *cellxgene* Atlas (33) to enhance the human and mouse networks and from the EBI Single Cell Expression Atlas to extend coverage to more model organisms (34). This expansion will provide more detailed, organism-specific co-expression maps, enabling more precise identification of gene co-expression patterns and cross-species comparisons, particularly for development, immune response and disease-related gene expression dynamics.

## Improved enrichment analysis

Gene set enrichment analysis is a critical component of high-throughput biological studies, providing key insights into the biological characteristics of datasets. This analysis is pivotal in identifying essential aspects such as biological processes, pathways and phenotypes, which are crucial for understanding the biological implications of experiments. Typically, overrepresentation analysis is employed, wherein a subset of data exceeding a specific cutoff (empirical or customary) is compared against an experimental background. By default, STRING uses the whole genome/proteome background; however, it is recommended that users provide a custom background list representing a more realistic universe of genes/proteins detected by their assay (35). Alternatively, the entire sorted dataset can be analyzed using gene set enrichment analysis to determine whether the distribution of genes at the top, bottom or both ends deviates significantly from what would be expected by chance. STRING supports both of these enrichment methods. The most recent update to STRING introduces various improvements, including a new false discovery rate (FDR) correction, enhanced filtering options and a new graphical visualization.

### FDR correction

In its analyses, STRING performs a test for each pathway (‘term’) within a given pathway collection (‘category’). Like many other enrichment tools, STRING employs the Benjamini–Hochberg correction (36) to adjust for multiple hypothesis testing. This statistical correction is essential for managing the FDR effectively. However, the larger the term count and the greater the diversity of the terms, the higher the required correction and the lower the chance of exceeding the alpha level (significance threshold) for a given term. One method used to address this issue involves restricting the analysis to a subset of terms, such as ‘GO slims’ that focus on broad, high-level terms with a simplified ontology structure (16). However, this approach has some disadvantages: it might exclude terms particularly relevant to a tested gene set, it may not cover all functions by design and it is mainly applicable to hierarchical classification systems such as the Gene Ontology.

STRING utilizes several hierarchical ontologies, such as the Brenda Tissue Ontology, Disease Ontology, Human Phenotype Ontology and the aforementioned Gene Ontology; they typically contain many more leaf-ward nodes than nodes closer to the root. The parental nodes must contain all the genes included in the child terms, creating an imbalance in the ontology with many more smaller terms and fewer larger terms. Removing smaller terms can substantially increase statistical power. However, simply eliminating all small terms could negatively impact discoverability, especially for small query gene sets where the smaller terms might be the most relevant for biological interpretation. The ontologies are usually large, with >10 000 terms (and therefore tests) in the Biological Process branch of the Gene Ontology tree. Such strong corrections have the effect that for larger query gene sets, or for backgrounds that cover only part of the proteome, it might not be statistically possible to detect enrichments for a subset of the terms; these can therefore be removed from consideration.

In its new version, STRING takes a more flexible approach—by only testing terms that have *a priori* any statistical potential for enrichment. This decision is based on several parameters: the size of the term, the size of the query set, the background size and the number of tests conducted. By calculating the smallest and largest term sizes that could theoretically be enriched given the FDR correction, STRING can determine which terms are viable for inclusion in the analysis. Terms outside these bounds are excluded from testing. This methodical exclusion significantly enhances the statistical power of the enrichment analysis, without omitting any terms that could be enriched. For smaller query sets, this strategy will maintain all terms, as even the smallest tested sets (term size = 2) might still be theoretically enriched. However, for larger, less specific sets or for tests against a custom smaller background, this approach markedly improves the statistical power, enabling STRING to perform more focused analyses without the drawbacks of increased false positive rates or omitting potentially biologically interesting terms. Although this method leverages the characteristics of ontologies, it does not require a hierarchy (parent–child relationships between the terms) to function and can be applied to any pathway/term collection with an imbalance of small terms, such as PMID-derived gene sets and STRING neighborhood clusters.

### Term filtering

One key insight from our online user surveys is that the often overwhelming number of enrichment results can make it challenging to discern the most relevant enriched terms. We have thus enhanced the display of enrichment results, allowing users to filter and sort them based on (i) FDR, (ii) strength, (iii) signal, (iv) term size and (v) term similarity.

The newest addition to our filtering options are the ‘term similarity’ and ‘signal’ filters. The ‘term similarity’ filter uses the Jaccard index to measure the similarity between the gene sets of terms within the same category. The method proceeds by sorting the terms according to their *P*-values, prioritizing those with the strongest statistical significance. The algorithm then iterates through the list of terms; any term that exhibits a similarity exceeding a predetermined, user-defined threshold relative to terms already included in the results is subsequently excluded. This approach requires no additional information beyond what is already available in the dataset and can be uniformly applied across all categories of terms. The ‘signal’ filter is defined as a weighted harmonic mean between the ratio of observed to expected gene occurrences in an enriched term and its −log(FDR), respectively. This filter balances the impact of both metrics and provides a more intuitive ordering of enriched terms and has been implemented as the default sorting criterion.

These filters are designed to exclude potentially less relevant results, such as terms that are marginally significant, small or show significant overlap in terms of gene content. This enhancement streamlines the search for relevant observations, helping users focus on the most meaningful results.

### Graphical visualization

In response to user feedback requesting more intuitive, graphical visualizations of the detected enrichments, STRING now has adopted an interactive dot plot as a primary display method for enrichment analysis outcomes. We chose the dot plot for its clarity in representing enriched functions across three dimensions: (i) enrichment signal along the *X*-axis, (ii) the FDR indicated through the color coding of the dots and (iii) the term’s protein count in the network represented by the size of each dot. The terms (listed on the *Y*-axis) are ranked by their enrichment signal or, if grouped by similarity, by the enrichment signal of their group first (Figure 2). This visualization reflects all filter settings chosen by the user to manage the volume of results displayed. These plots can be additionally customized in the interface and the resulting graphs can be downloaded as PNG for immediate use or as SVG for further modifications.

Furthermore, a distinctive feature of STRING’s visualization is the incorporation of similarity groups, which visually group related terms on the plot, adding an additional dimension to the enrichment results. These groups utilize computed similarities to cluster terms together, highlighting relationships between the terms and aiding the analysis. The clustering employs the average linkage method based on the Jaccard index, with a user-specified cutoff applied to form groups that, on average, share the specified similarity. The groups are sorted by the maximum signal of their terms, with each term within a group subsequently sorted by its individual signal. This grouping is visually highlighted on the right side of the plot.

## STRING clustering and gene set descriptions

STRING offers network clustering options for user-submitted gene lists. This feature visually connects nodes that are more interconnected to each other than to other nodes, reflecting the functional modularity of the user’s gene list. By grouping proteins that closely interact, clustering aids in the discovery of functional modules and facilitates hypothesis generation. Clustering is based on the connectivity between nodes, incorporating edge weights (combined scores) reflecting the confidence level of the interactions. Only visible edges—those corresponding to active channels with confidence higher than the user-specified cutoff—are considered, ensuring that clustering is consistent with the network being shown.

Users have the choice of two different clustering algorithms. ‘*K*-means clustering’ is implemented via the *k*-medoids algorithm [pyclustering package (37)], allowing users to specify the desired number of clusters (*k*). This method forms clusters based on a distance matrix derived from the most probable paths between nodes. In case of multiple disconnected components within the network, STRING aims for a balanced distribution of cluster sizes by iteratively dividing the largest components first. In contrast, ‘MCL clustering’, implemented with standalone binaries (release 14-137), automatically generates natural divisions based on the network’s inherent structure. It utilizes the Markov cluster algorithm (38) to simulate stochastic flow in graphs, identifying groups of highly interconnected nodes as clusters based on their combined STRING scores. The user-controlled inflation parameter influences the granularity of the clustering. This method is particularly effective in capturing the true modular nature of biological networks, allowing for intuitive groupings that correspond to biological functions and relationships.

Researchers often analyze the biological context of the obtained clusters to interpret and draw conclusions from network data. To aid this process, we have developed a novel gene set naming feature that significantly enhances the interpretability of cluster analyses. This feature automatically assigns the best description for each cluster based on STRING’s robust statistical enrichment analysis, simplifying the often cumbersome task of manually interpreting cluster data. It ranks the enriched terms by their enrichment signal (see the ‘Improved enrichment analysis’ section), choosing the primary, secondary and tertiary names among various enrichment categories. This prioritization ensures that the names reflect the most statistically significant and biologically pertinent attributes of each cluster, providing a clear, immediate understanding of its functional characteristics. The enrichment primarily draws from categories such as Gene Ontology Biological Processes, while incorporating a range of other categories for broader annotations. In case the enrichment analysis yields no significant results or if the cluster contains only one gene, STRING assigns canonical gene names as descriptions to ensure that clusters are easily identifiable. This functionality extends beyond cluster analysis and is applicable to any gene set. As such, it is also available through an API, complementing our suite of other API methods. The new API function, named *geneset_description*, requires only a set of genes as input and automatically performs enrichment analysis. It returns up to three descriptions—primary, secondary and tertiary—based on relevance and availability, identical to the cluster naming in the user interface. These descriptions are filled sequentially, with the ‘primary’ always representing the most relevant term. One application of this API is in the stringApp Cytoscape plugin, which utilizes it to automatically overlay the assigned descriptions onto each network cluster after clustering is executed.

## Network and protein embeddings

To facilitate the use of STRING in machine learning applications, users can now directly download precomputed ProtT5 (39) sequence and cross-species protein network embeddings derived from STRING, for all eukaryotes, and utilize the embeddings on their own labeled datasets. The embeddings encode information from two aspects. Sequence embeddings can capture protein domains and shorter sequence motifs, while network embeddings can complement these with information on cellular organization such as protein complexes and pathways that may not be evident from sequence data alone.

While protein sequence embeddings are inherently comparable across species due to the universal nature of amino acid sequences, the primary obstacle to using network embeddings is ensuring that network embeddings from different species are directly comparable. This challenge arises because protein networks are independent for each species, and traditional network embedding methods are not designed to address cross-species comparability. The lack of comparability between network embeddings prevents the effective transfer of knowledge and findings across different species. To address this, we developed a method to align eukaryotic network embeddings across species using orthologous relationships based on FedCoder (40). This alignment technique creates a unified embedding space where proteins from different eukaryotic species can be directly compared. As a result, it enhances cross-species protein predictions, particularly in tasks such as subcellular localization and function prediction.

In the protein machine learning field, having precomputed sequence embeddings and cross-species compatible network embeddings from STRING enables researchers to use these resources directly, eliminating the need to calculate embeddings themselves. This strategy not only reduces the energy footprint resulting from redundant calculations across research groups, but also enhances reproducibility in computational biology research promoting more sustainable and consistent scientific practices.

## Data Availability

The STRING database is freely available online at https://string-db.org/.
